# Improved clinical outcomes of patients with ovarian carcinoma arising in endometriosis

**DOI:** 10.18632/oncotarget.13967

**Published:** 2016-12-15

**Authors:** Jiaqi Lu, Xiang Tao, Jiayi Zhou, Yingying Lu, Zehua Wang, Haiou Liu, Congjian Xu

**Affiliations:** ^1^ Department of Gynaecology, Obstetrics and Gynecology Hospital, Fudan University, Shanghai, China; ^2^ Shanghai Key Laboratory of Female Reproductive Endocrine Related Diseases, Obstetrics and Gynecology Hospital, Fudan University, Shanghai, China; ^3^ Department of Pathology, Obstetrics and Gynecology Hospital, Fudan University, Shanghai, China

**Keywords:** ovarian cancer, endometriosis, overall survival, progression-free survival, prognostic marker

## Abstract

**Background:**

Despite enormous efforts to dissect the role of endometriosis in ovarian cancer development, the difference in prognosis between ovarian cancer patients with or without endometriosis remains elusive. The purpose of this study is to assess the association between endometriosis and the prognosis in patients with ovarian cancer.

**Results:**

Ovarian cancer arising in endometriosis tended to be presented as clear cell histology, early stage, less intraperitoneal metastasis and ascites, and lower CA125 level compared with those without endometriosis. Multivariate Cox regression analysis identified endometriosis as an independent prognostic factor for progression free survival (P = 0.002) and overall survival (P = 0.009) in all patients and especially for early stage. A nomogram integrating endometriosis, FIGO stage and CA125 was established to predict progression free survival and overall survival.

**Materials and methods:**

This study retrospectively enrolled 196 ovarian cancers arising or not in endometriosis judged by adjunctive use of CD10 immunohistochemistry in conjunction with H&E staining specimens. Clinicopathologic variables, progression-free survival (PFS) and overall survival (OS) were recorded. Kaplan-Meier analysis was performed to compare survival curves. Cox regression models were used to analyze the effect of endometriosis on PFS and OS. A prognostic nomogram was constructed based on the independent prognostic factors identified by multivariate analysis.

**Conclusions:**

Endometriosis is an independent predictor of prognosis in ovarian cancer patients.

## INTRODUCTION

Endometriosis, a chronic gynecological disease, shares common characteristics with malignant cells [1]. Although endometriosis remains largely benign, malignant transformation may account for up to 1% of cases, most commonly from ovarian lesions [2, 3]. In addition to epidemiological evidence between endometriosis and ovarian cancer, the pathological findings confirmed endometriosis in close proximity to the tumor [4, 5]. Both ovarian clear cell and endometrioid carcinoma are associated with endometriosis [6].

The difference in prognosis between ovarian cancer patients with and without endometriosis remains elusive. Previous studies reported that ovarian cancer patients with endometriosis are associated with better prognosis compared with those without concomitant endometriosis [7–10]. However, other studies have not confirmed these findings after adjusting for potential confounding factors [11]. Resolving this issue is difficult because the criteria for the diagnosis of endometriosis associated ovarian cancer (EAOC) is heterogenous. Given these conflicting findings, we sought to characterize ovarian cancers arising from endometriosis based on pathological identification and to evaluate the prognostic impact of the endometriosis on ovarian cancer patients for risk stratification.

## RESULTS

### Patient characteristics and associations with endometriosis

**Figure 1:**
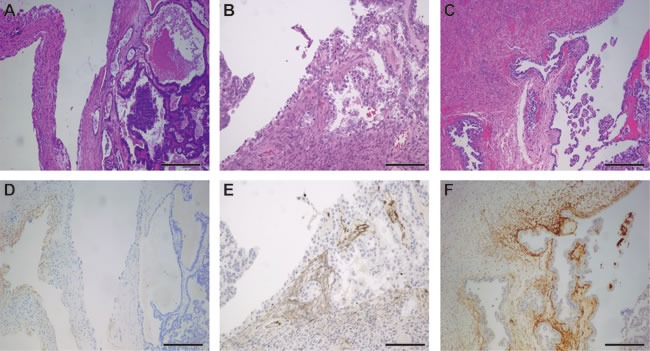
Representative photographs of ovarian clear cell carcinoma arising in endometriosis **A.-C.** H&E staining of ovarian clear cell carcinoma arising in endometriosis. **D.-F.** Weak, moderate and strong immunohistochemical staining with CD10 of stroma in ovarian clear cell carcinoma specimen. Bar = 100μm.

A total of 196 patients met the inclusion criteria, of which 58 (30.0%) cases have been affected by tumors arising in endometriosis, while 138 (70.0%) had no concomitant endometriosis. Of the 58 specimens were histologically positive for ovarian cancer arising in endometriosis by H&E staining, reconfirmation of all samples by CD10 staining. CD10 IHC result was positive in each endometriosis specimens judged by H&E staining (Figure 1). CD10 staining was confined to endometrial stromal cells, and generally moderate to strong (Supplementary Table 1). Patients and tumor characteristics of the two groups are presented in Table 1. Forty-eight patients (82.76%) were clear cell histology type arising from endometriosis compared with 65.94% of clear cell ovarian cancer without endometriosis (P = 0.048). Fifty-one patients (87.93%) with endometriosis were diagnosed at the FIGO stage (I-II) compared with 66.67% of patients without endometriosis (P = 0.004). Intraperitoneal metastasis was detected in 41 patients (29.71%) in the endometriosis-free group, compared to 8 (13.79%) patients with endometriosis group (P = 0.03). Twenty-one patients (15.22%) without endometriosis tend to have more ascites compared to 1 case (1.42%) arising in endometriosis (P = 0.013). Eighty-three patients (60.14%) without endometriosis present high CA125 level, compared to 25 patients (43.1%) arising in endometriosis (P = 0.042). No association between endometriosis and other clinicopathologic characteristics was observed.

**Table 1:** Patient characteristics in ovarian cancer arising or not from endometriosis

Characteristic	Endometriosis		*P* value
	No (n =138)	Arising (*n* = 58)	
Age (years)	51.08 (49.45-52.71)	49.64(47.62-51.65)	0.314
Histology Clear cell Endometrioid Mixed	91 (65.94%) 37 (26.81%) 10 (7.25%)	48 (82.76%) 9 (15.52%) 1 (1.72%)	**0.048**
Ovarian involvement Monolateral Bilateral	108 (78.26%) 30 (21.74%)	52 (89.66%) 6 (10.34%)	0.060
ECOG performance status 0-1 2-3	128 (92.75%) 10 (7.25%)	55 (94.83%) 3 (5.17%)	0.827
FIGO stage I II III IV	75 (54.35%) 17 (12.32%) 40 (28.99%) 6 (4.35%)	46 (79.31%) 5 (8.62%) 7 (12.07%) 0 (0.00%)	**< 0.001**
FIGO stage Early stage (I/II) Late stage (III/IV)	92 (66.67%) 46 (33.33%)	51 (87.93%) 7 (12.07%)	**0.004**
Lymph node metastasis negative positive	121 (87.68%) 17 (12.32%)	54 (93.10%) 4 (6.90%)	0.386
Intraperitoneal metastasis negative positive	97 (70.29%) 41 (29.71%)	50 (86.21%) 8 (13.79%)	**0.030**
Residual tumor (cm) ≤ 1 > 1	121 (87.68%) 17 (12.32%)	54 (93.10%) 4 (6.90%)	0.386
Preoperative ascites (ml) < 500 ≥ 500	117 (84.78%) 21 (15.22%)	57 (98.28%) 1 (1.42%)	**0.013**
Preoperative CA125 level (U/ml) < 35 ≥ 35	55 (39.86%) 83 (60.14%)	33 (56.90%) 25 (43.10%)	**0.042**

Abbreviations: ECOG, Eastern Cooperative Oncology Group; FIGO, International federation of gynecology and obstetrics; CA125, cancer antigen 125. All data presented as median (95% CI) or number. Bold values indicate P < 0.05.

### Endometriosis is associated with PFS and OS in ovarian cancer patients

**Figure 2:**
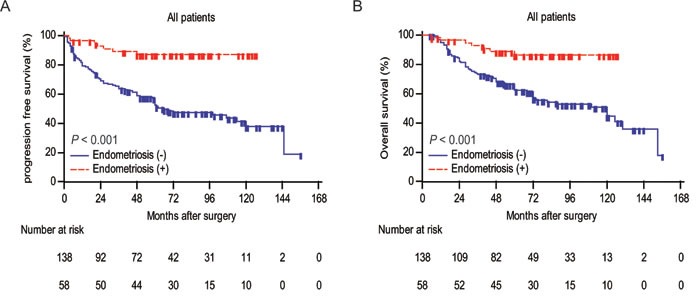
Analyses of progression-free survival and overall survival according to endometriosis in all patients **A.** Kaplan-Meier curves for PFS of ovarian cancer patients categorized by endometriosis. Patients who were lost to follow-up or who showed no progression at the time of the last follow-up were censored (+). **B.** Kaplan-Meier curves for OS of ovarian cancer patients categorized by endometriosis. Patients who were lost to follow-up or who were still alive at the time of the last follow-up were censored (+). P values were calculated by log-rank test.

**Figure 3:**
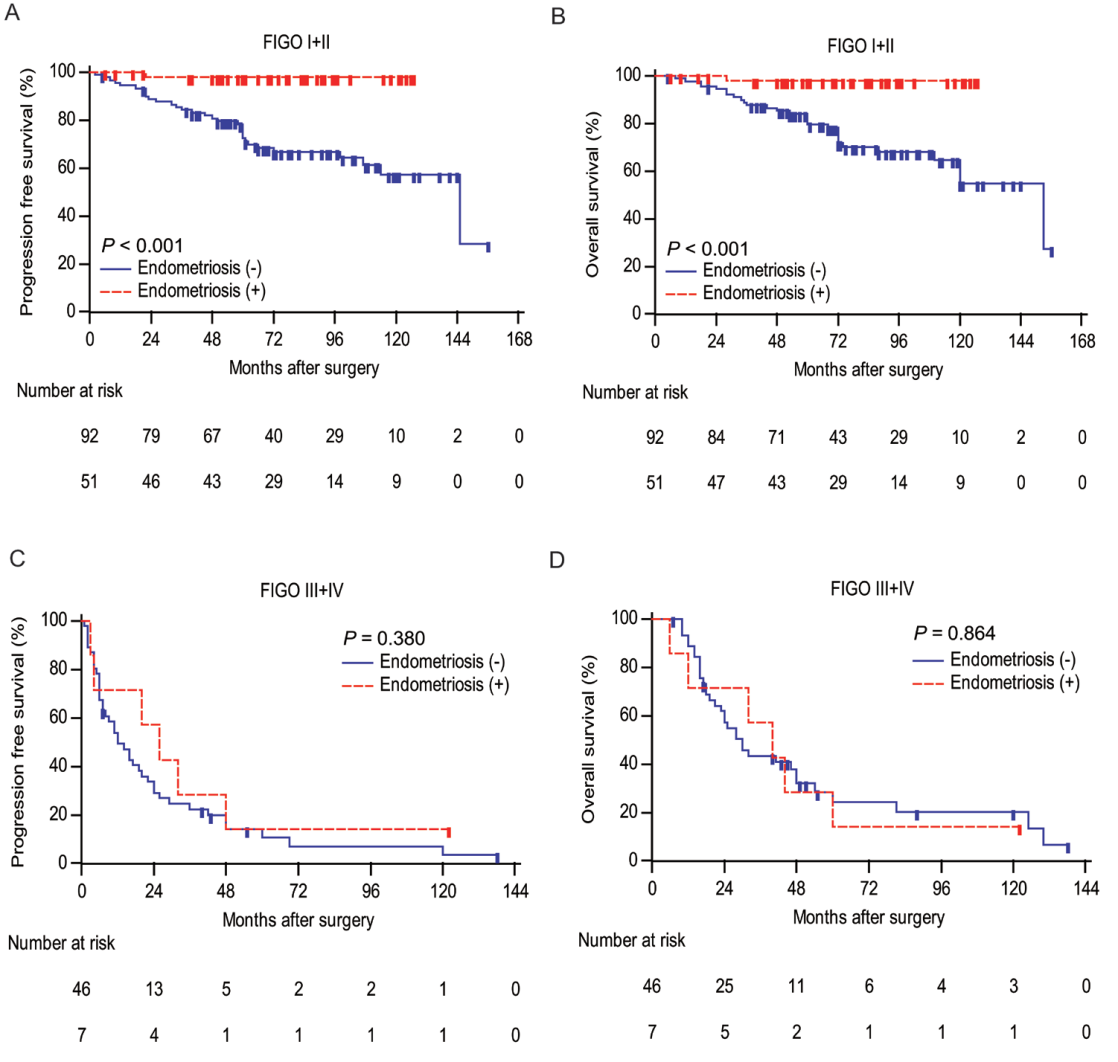
Analyses of progression-free survival and overall survival according to endometriosis in different FIGO stage groups **A., C.** Kaplan-Meier curves for PFS of ovarian cancer patients categorized by endometriosis in FIGO stage I+II and III+IV, respectively. Patients who were lost to follow-up or who showed no progression at the time of the last follow-up were censored (+). **B., D.** Kaplan-Meier curves for OS of ovarian cancer patients categorized by endometriosis in FIGO stage I+II and III+IV, respectively. Patients who were lost to follow-up or who were still alive at the time of the last follow-up were censored (+). P values were calculated by log-rank test.

To further estimate the relationship between endometriosis and clinical outcomes of ovarian cancer patients, we applied Kaplan-Meier survival analysis and log-rank test to compare PFS and OS between two groups. As shown in Figure 2, patients with endometriosis were significantly associated with late recurrence (P < 0.001) and better OS (P < 0.001). We further performed a subgroup analysis by FIGO stage (Figure 3). The prognostic value of endometriosis is more prominent in patients with early stage (FIGO I-II) (P < 0.001 for both PFS and OS).

### Endometriosis is an independent predictor of PFS and OS

To determine the prognostic significance of clinicopathologic variables of PFS and OS, we performed univariate Cox analysis. As present in Table 2, endometriosis was identified as a protective factor that might affect PFS (hazard ratio (HR) 0.187, P < 0.001) and OS (HR 0.238, P < 0.001) of ovarian cancer patients. In addition, FIGO stage, lymph node metastasis, intraperitoneal metastasis, residual tumor, ascites, and CA125 were identified as unfavorable factor for PFS and OS. On multivariate analysis, endometriosis is an independent prognostic factor for PFS (HR 0.284, P = 0.002) and OS (HR 0.349, P = 0.009).

**Table 2:** Univariate and multivariate Cox regression analysis for progression-free survival (PFS) and overall survival (OS) of ovarian cancer patients according to various clinic-pathologic factors (n = 196)

Clinical variables	PFS		OS	
	HR (95%CI)	*P* value	HR (95%CI)	*P* value
**Univariate analysis**				
Age	0.999 (0.975-1.023)	0.915	1.003 (0.977-1.030)	0.814
Histology clear cell Endometrioid Mixed	Reference 0.967 (0.585-1.671) 1.896 (0.903-3.982)	0.286	Reference 0.910 (0.514-1.611) 1.845 (0.867-3.926)	0.282
ECOG performance 0-1 2-3	Reference 1.895 (0.913-3.932)	0.088	Reference 1.567 (0.678-3.621)	0.296
FIGO stage Early stage (I-II) Late stage (III-IV)	Reference 10.367 (6.460-16.638)	**<0.001**	Reference 7.413 (4.526-12.139)	**<0.001**
Lymph node metastasis negative positive	Reference 4.707 (2.786-7.952)	**<0.001**	Reference 3.048 (1.684-5.517)	**<0.001**
Intraperitoneal metastasis negative positive	Reference 6.182 (3.908-9.778)	**<0.001**	Reference 5.194 (3.213-8.398)	**<0.001**
Residual tumor (cm) ≤ 1 > 1	Reference 5.639 (3.375-9.423)	**<0.001**	Reference 4.556 (2.651-7.831)	**<0.001**
Preoperative ascites (ml) < 500 ≥ 500	Reference 2.914 (1.682-5.047)	**<0.001**	Reference 2.653 (1.473-4.779)	0.001
Preoperative CA125 (U/ml) < 35 ≥ 35	Reference 2.524 (1.561-4.079)	**<0.001**	Reference 2.570 (1.536-4.301)	**<0.001**
Endometriosis negative positive	Reference 0.187 (0.086-0.405)	**<0.001**	Reference 0.238 (0.109-0.518)	**<0.001**
**Multivariate analysis**				
FIGO stage Early stage (I-II) Late stage (III-IV)	Reference 8.642 (5.339-13.988)	**<0.001**	Reference 6.158(3.732-10.163)	**<0.001**
Preoperative CA125 (U/ml) < 35 ≥ 35	Reference 2.033 (1.249-3.309)	**0.003**	Reference 2.150 (1.275-3.626)	**0.010**
Endometriosis negative positive	Reference 0.284 (0.130-0.623)	**0.002**	Reference 0.349 (0.159-0.765)	**0.009**

Abbreviations: ECOG, Eastern Cooperative Oncology Group; FIGO, International federation of gynecology and obstetrics; CA125, cancer antigen 125; HR, hazard ratio; 95% CI, 95% confidence interval.

Bold values indicate P < 0.05.

### Association between endometriosis and clinical outcomes in early stage patients

To evaluate the clinical usefulness of endometriosis in early stage ovarian cancer, we performed subgroup analysis upon early stage patients. By univariate analysis, intraperitoneal metastasis (P < 0.001 for both PFS and OS) and residual tumor (P = 0.027, P = 0.021 for PFS and OS, respectively) were significantly associated with poor clinical outcomes, while endometriosis (P = 0.004, P = 0.007 for PFS and OS, respectively) was significantly associated with better prognosis (Table 3). Multivariate analysis showed that endometriosis remained as an independent indicator of PFS (HR 0.054, P = 0.004) and OS (HR 0.064, P = 0.007).

**Table 3:** Univariate and multivariate Cox regression analysis for progression-free survival (PFS) and overall survival (OS) of ovarian cancer patients with FIGO stage (I/II) according to various clinic-pathologic factors (n=143)

Clinical variables	PFS		OS	
	HR (95%CI)	*P* value	HR (95%CI)	*P* value
**Univariate analysis**				
Age	1.009 (0.971-1.049)	0.643	1.024 (0.982-1.067)	0.273
Histology clear cell Endometrioid Mixed	Reference 1.525 (0.701-3.317) 3.891 (1.315-11.511)	0.085 0.290 **0.015**	Reference 1.252 (0.538-2.915) 3.577 (1.202-10.646)	0.136 0.604 **0.023**
ECOG performance 0-1 2-3	Reference 2.370 (0.828-6.785)	0.110	Reference 1.222 (0.290-5.154)	0.786
Intraperitoneal metastasis negative positive	Reference 4.938 (2.171-11.234)	**<0.001**	Reference 4.710 (1.960-11.320)	**<0.001**
Residual tumor (cm) ≤ 1 > 1	Reference 5.092 (1.215-21.341)	**0.027**	Reference 5.494 (1.301-23.196)	**0.021**
Preoperative ascites (ml) < 500 ≥ 500	Reference 1.942 (0.681-5.538)	0.217	Reference 1.480 (0.448-4.895)	0.522
Preoperative CA125 (U/ml) < 35 ≥ 35	Reference 1.813 (0.890-3.694)	0.103	Reference 1.825 (0.864-3.853)	0.117
Endometriosis negative positive	Reference 0.054 (0.007-0.395)	**0.004**	Reference 0.062 (0.009-0.455)	**0.007**
**Multivariate analysis**				
Intraperitoneal metastasis negative positive	Reference 5.049 (2.213-11.517)	**<0.001**	Reference 4.566 (1.899-10.976)	**<0.001**
Endometriosis negative positive	Reference 0.054 (0.007-0.391)	**0.004**	Reference 0.064 (0.009-0.462)	**0.007**

Abbreviations: ECOG, Eastern Cooperative Oncology Group; FIGO, International federation of gynecology and obstetrics; CA125, cancer antigen 125; HR, hazard ratio; 95% CI, 95% confidence interval.

Bold values indicate P < 0.05.

### Construction and validation of prognostic nomogram for PFS and OS

**Figure 4:**
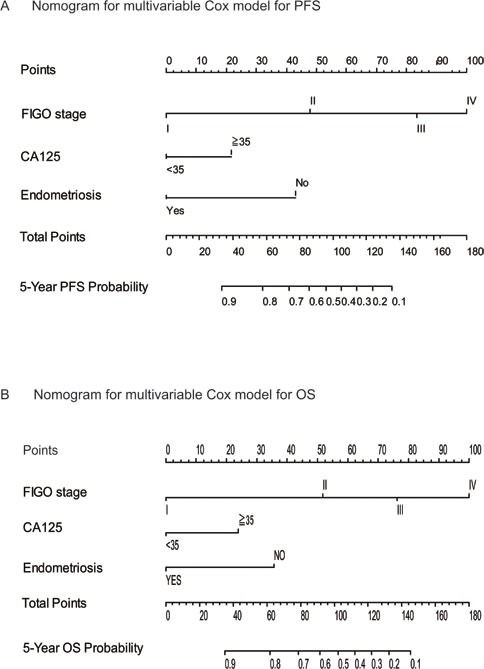
Survival nonograms Nomograms were created from the multivariable Cox model. The presence or absence of each variable is scored (top row). The cumulative score from each variable is used to calculate 5-year PFS **A.** and OS **B.** probabilities. PFS = progression-free survival. OS = overall survival.

**Figure 5:**
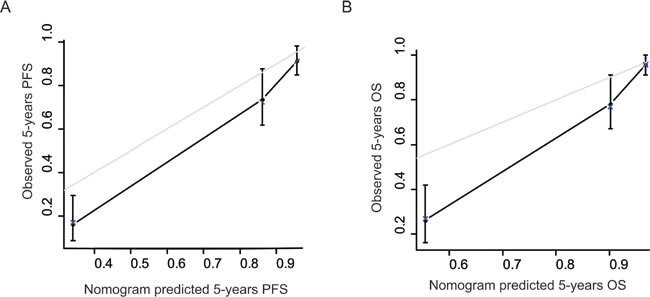
The calibration curves for predicting patient survival at each time point Nomogram-predicted 5-year PFS **A.** and 5-year OS **B.** is plotted on the x-axis; actual PFS and OS is plotted on the y-axis. A plot along the 45-degree line would indicate a perfect calibration model in which the predicted probabilities are identical to the actual outcomes.

Significant prognostic factors were concluded from multivariate Cox regression analysis of PFS and OS to establish nomogram (Figure 4). The nomogram illustrated FIGO stage as sharing the largest contribution to prognosis, followed by endometriosis and CA125. The calibration plot for the nomogram presented an optimal agreement between the predicted and actual observation for the PFS and OS at 5-year (Figure 5).

## DISCUSSION

Our study reported endometriosis as an independent predictor for PFS and OS in patients with ovarian cancer. Moreover, endometriosis manifests a discriminative power in early stage ovarian cancer subgroups, which can help guiding management of patients with early FIGO stage. The nomogram integrating endometriosis, FIGO stage and CA125 predicts 5-year PFS and OS well for ovarian cancer patients.

Ryu et al. explored most of the patients had diagnosed at an early stage (stage I, 61.3%), and the overall 5-year survival rate was 57% [12]. Consistently, in our cohort, 121 (61.7%) out of all patients was diagnosed at FIGO stage I, and the overall 5-year survival rate was 69.5%. Moreover, we found that the overall 5-year survival rate was 91.5% in stage I, 56.3% in stage II, 26.4% in stage III, and 0% in stage IV (Supplementary Figure 1), which is quite consistent with data from the United states [13]. However, our study demonstrated that patients of ovarian cancer arising from endometriosis had a better prognosis, with 86.6% 5-year survival compared with 62.4% in patients of ovarian cancer without endometriosis, which was consistent with Shuang et al. [14].

The criteria for the definition of endometriosis associated ovarian cancer varied between different studies. Some authors considered the tumors as EAOC on the basis of malignant transformation in the endometriosis glands leading to carcinoma [10], whereas others included cases if either the transition point was identified or merely in the setting of any endometriosis was found within the surgical specimen coexisting with cancer [15]. We adopted the strictly histologic criteria for the diagnosis of ovarian cancer arising in endometriosis [16]. Additionally, the use of CD10 immunohistochemistry confirmed the diagnoses of ovarian cancer arising from endometriosis [17].

In our data, when FIGO stage was controlled, endometriosis remains significant on multivariate analysis between subjects and controls. This supports the hypothesis that EAOC as a distinct disease from non-EAOC with better prognosis [18]. ARID1A (AT-rich interactive domain 1A) mutations and consequent loss of BAF250a (BRG-associated factor 250a) protein expression were particularly identified in EAOC, and in the contiguous atypical endometriotic lesions, but not in distinct endometrioid lesions far from the carcinoma, suggesting such phenomenon as a possible early event in the malignant transformation of endometriosis [19, 20]. ARID1A mutations induce chromatin remodeling dysfunction and tend to coexist with activating PIK3CA mutations [21]. Moreover, atypical endometriosis and EAOC may share several molecular alterations such as ARID1A and PIK3CA mutations, PTEN loss, MET amplifications, and HNF1B overexpression, suggesting a common molecular mechanism for malignant development [22–24]. In an Apc- and Pten-defective mouse ovarian cancer model loss of ARID1A enhances epithelial differentiation and prolongs survival, which may account for the better prognosis of ovarian cancer arising in endometriosis [25]. The clinical significance of loss of ARID1A in EAOC has remained to be elucidated.

Our data are consistent with that of Shuang et al [14], who reported that 78.5% of clear cell cancer associated with endometriosis presented as stage I and II compared with 37.4% of clear cell ovarian cancers that are not associated with endometriosis. With regard to endometriosis as independent predictor for prognosis, there was a discrepancy between the study of Shuang et al [14] and our own. Resolving this issue only be these two studies is difficult because the number of patients in both studies was insufficient. Potential limitation of this analysis should be considered. This is a retrospective analysis, and data were obtained by clinical records; thus, only hard information, such as age and stage of the disease, were collected.

In conclusion, our study revealed that endometriosis is associated with better clinical outcomes in ovarian cancer patients. A prognostic model integrating endometriosis, FIGO stage and CA125 may improve the management of ovarian cancer patients in terms of risk stratification, individualizing postsurgical follow-up.

## MATERIALS AND METHODS

### Patients

After approval from institutional review board, we retrospectively identified 196 cases with a primary diagnosis of pure clear cell, endometrioid, or mixed ovarian cancer at our hospital between 1995 and 2010. Of the mixed tumors, 8 cases showed clear cell histology with endometrioid histology, whereas the remaining tumors showed heterogeneous histology consisting of endometrioid, clear cell, and serous differentiation. All patients underwent surgery according to Federation of Gynecology and Obstetrics (FIGO) guidelines for ovarian cancer. All patients received a platinum-based chemotherapy regimen, and the number of cycles ranged from six to nine after tailoring to different individuals. Microscopic slides were reviewed and confirmed by two experienced gynecologic pathologist (Dr. XT and JZ). Patients were divided into two groups according to the detection of cancer arising from ovarian endometriosis or not on the basis of the Sampson and Scott criteria: 1) the presence of both benign and neoplastic endometrial tissues in the tumor, 2) histological findings compatible with an endometrial origin, 3) the discovery of no other primary tumor sites, and 4) a morphologic demonstration of a continuum between benign and malignant epithelium [10]. Patients with the pathological findings confirmed endometriosis in close proximity to the tumor, but not histologically contiguous to the ovarian cancer tissue were excluded. The inclusion and exclusion criteria were summarized in Supplementary Figure 2. No statistically significant differences in tumor characteristics or survival outcomes were observed for included and excluded patients (Supplementary Table 2 and Supplementary Figure 3).

### Immunohistochemistry

The primary formalin-fixed, paraffin-embedded ovarian cancer tissues arising from endometriosis were applied with CD10 immunohistochemical staining. Immunohistochemistry protocol was described previously [26]. The primary antibody against human CD10 (clone 56C6, DAKO; dilution: 1: 200) was applied in the procedure. Positive staining was subjectively classified as weak, moderate, or strong.

### Statistical analysis

Correlations between endometriosis and clinicopathologic characteristics were analyzed with χ2 test. Clinical outcomes were assessed by progression free survival (PFS) and overall survival (OS). PFS was defined as the interval between the date of surgery and the date of diagnosis of any type of progression. OS was defined as the interval between surgery and death. Patients were censored if they were lost to follow-up or if they show not progression or were still alive at the time of last follow-up. Follow up was updated in Feb 2015. Kaplan-Meier method with log-rank test was applied to compare survival curves. Univariate and multivariate Cox regression models were fitted to evaluate the effect of prognostic factors on OS and PFS, and P > 0.10 was the removal criterion when performing backward stepwise variable deletions. Nomogram was constructed as the prognostic model whose accuracy was evaluated by the Calibration plot. Data were analyzed using MedCalc software (version 12.7.0.0; MedCalc, Mariakerke, Belgium), and R software, version 3.1.2 (The R Foundation for Statistical Computing, http://www.r-project.org). All statistical tests were two sided and performed at a significance level of 0.05.

## SUPPLEMENTARY MATERIALS FIGURES AND TABLES


