# Supplementary material for: Improved clinical outcomes of patients with ovarian carcinoma arising in endometriosis
Source: Oncotarget. 2016 Dec 15;8(4):5843–52. doi: 10.18632/oncotarget.13967 (PMC5351594; doi:10.18632/oncotarget.13967)
Supplement: Supplementary file 1 [file oncotarget-08-5843-s001.pdf]

Improved clinical outcomes of patients with ovarian carcinoma arising in endometriosis

Supplementary Material

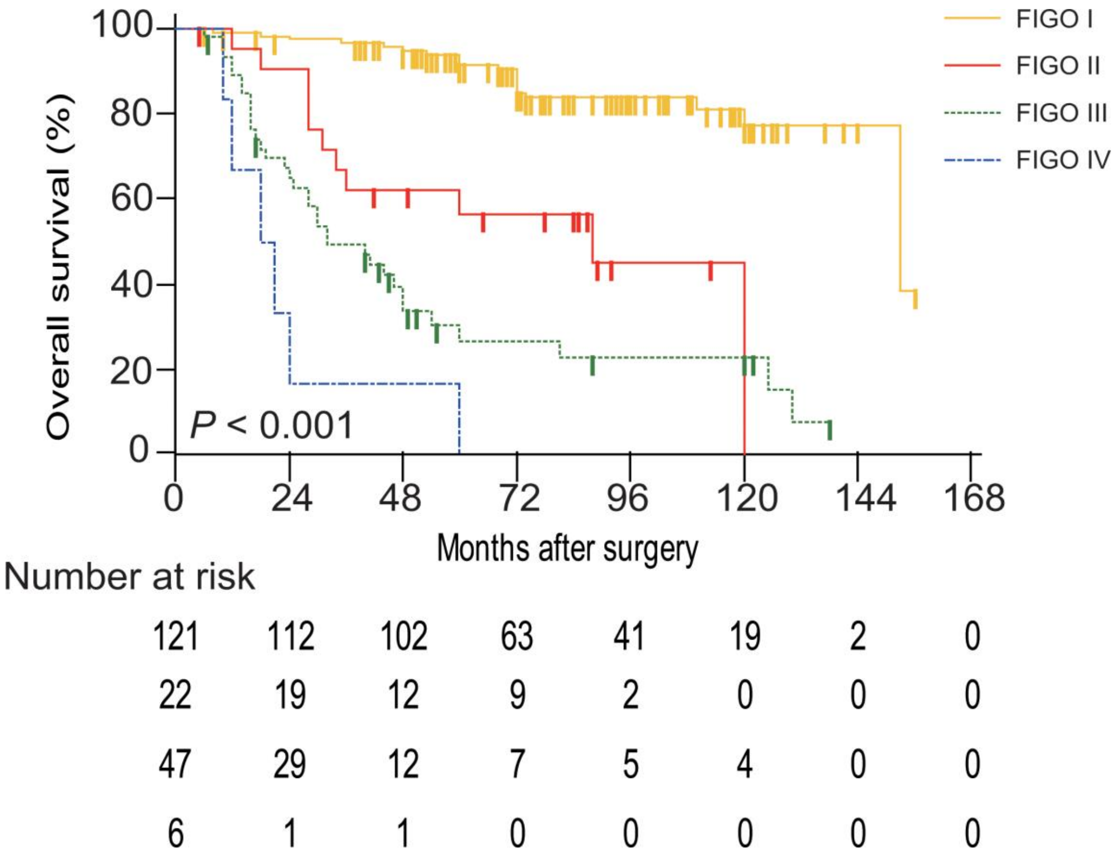

Supplementary Figure 1: **Analyses of overall survival according to FIGO stage in all patients.** Kaplan-Meier curves for OS of ovarian cancer patients categorized by FIGO stage I-IV. Patients who were lost to follow-up or who showed no progression at the time of the last follow-up were censored (+). *P* values were calculated by log-rank test.

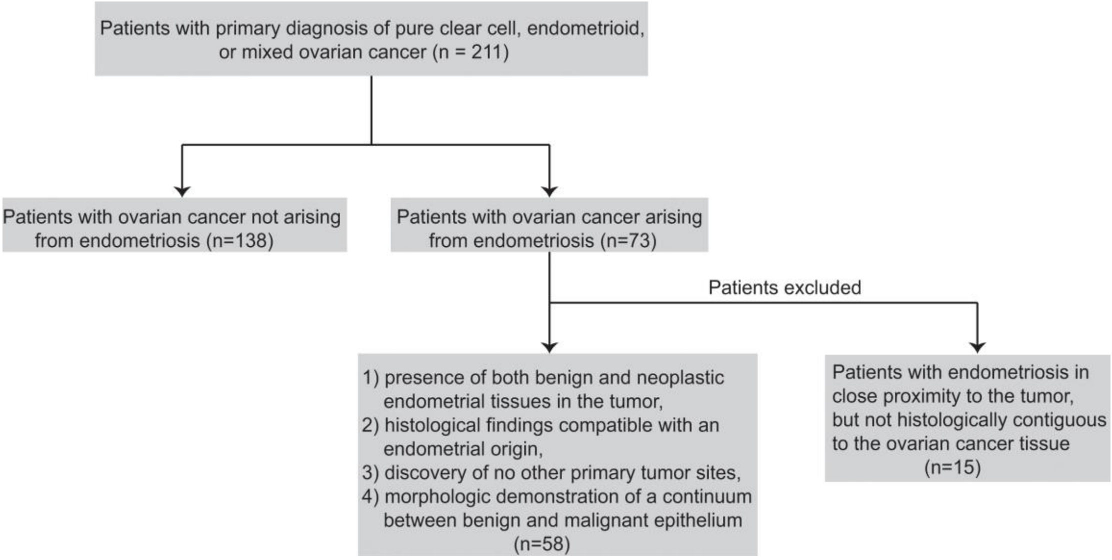

Supplementary Figure 2: Consort diagram outlining patient selection and exclusion criteria.

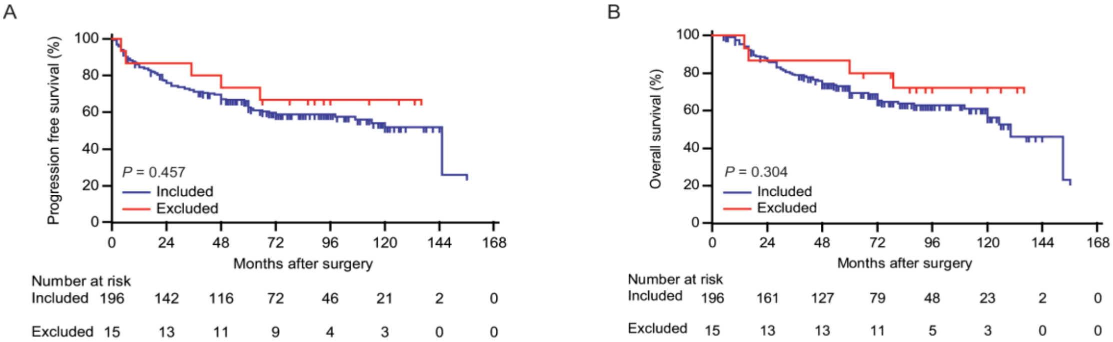

Supplementary Figure 3: Analyses of progression-free survival and overall survival according to patients categorized by selection criteria. (A)Kaplan-Meier curves for PFS of ovarian cancer patients categorized by selection criteria. Patients who were lost to follow-up or who showed no progression at the time of the last follow-up were censored (+). (B) Kaplan-Meier curves for OS of ovarian cancer patients categorized by selection criteria. Patients who were lost to follow-up or who were still alive at the time of the last follow-up were censored (+). *P* values were calculated by log-rank test.

Supp Table 1: CD10 IHC results in ovarian cancer specimens arising in endometriosis.

Histology	Number	CD10 IHC staining		
		Strong	Moderate	Weak
Clear cell	48	30	12	6
Endometrioid	9	6	3	0
Mix	1	1	0	0

Supplementary Table 2: Excluded data analysis

Characteristic	Included (n =196)	Excluded (n = 15)	*P* value
Age (years)	50.65 (49.37-51.94)	49.93(46.65-54.22)	0.767
Histology			0.439
Clear cell	139 (70.92%)	9 (60.00%)	
Endometrioid	46 (23.47%)	4 (29.67%)	
Mixed	11 (5.61%)	2 (13.33%)	
Ovarian involvement			0.478
Monolateral	160 (81.63%)	14 (93.33%)	
Bilateral	36 (18.37%)	1 (6.67%)	
ECOG performance status			0.289
0-1	183 (93.37%)	13 (86.67%)	
2-3	13 (6.63%)	2 (13.33%)	
FIGO stage			0.737
I	121 (61.73%)	10 (66.67%)	
II	22 (11.22%)	1 (6.67%)	
III	47 (23.98%)	3 (20.00%)	
IV	6 (3.06%)	1 (6.67%)	
FIGO stage			0.975
Early stage (I/II)	143 (72.96%)	11 (73.33%)	
Late stage (III/IV)	53 (27.04%)	4 (26.67%)	
Lymph node metastasis			0.601
negative	175 (89.29%)	14 (93.33%)	
positive	21 (10.71%)	1 (6.67%)	
Intraperitoneal metastasis			0.658
negative	147 (75.00%)	12 (80.00%)	
positive	49 (25.00%)	3 (20.00%)	
Residual tumor (cm)			0.760
≤ 1	175 (89.29%)	13 (86.67%)	
> 1	21 (10.71%)	2 (13.33%)	
Preoperative ascites (ml)			0.561
< 500	174 (88.78%)	14 (93.33%)	
≥ 500	22 (11.22%)	1 (6.67%)	
Preoperative CA125 level (U/ml)			0.713
< 35	88 (44.90%)	6 (40.00%)	
≥ 35	108 (55.10%)	9 (60.00%)	

Abbreviations: ECOG, Eastern Cooperative Oncology Group; FIGO, International federation of gynecology and obstetrics; CA125, cancer antigen 125. All data presented as median (95% CI) or number. Bold values indicate $P < 0.05$.
